# Adult Primary Hemophagocytic Lymphohistiocytosis (HLH) Initially Suspected as Brucellosis-Associated HLH: A Case Report

**DOI:** 10.7759/cureus.109793

**Published:** 2026-05-28

**Authors:** Hamad Alrasheedi, Ahmed H Alreshidi, Hani Almutairi, Salma Altamimy, Othman M Alodayli, Eman M Nassef

**Affiliations:** 1 Internal Medicine, King Salman Specialist Hospital, Ha'il, SAU; 2 Internal Medicine, King Khalid Hospital, Ha'il, SAU; 3 Internal Medicine, King Salman Specialist Hospital, Ḥaʼil, SAU; 4 Medicine and Surgery, University of Hail College of Medicine, Ha'il, SAU; 5 Internal Medicine, Hail Health Cluster, Hai'l, SAU; 6 Internal Medicine, Al-Azhar University, Cairo, EGY

**Keywords:** brucellosis, hscore, hyperferritinemia, pancytopenia, primary hemophagocytic lymphohistiocytosis, secondary hemophagocytic lymphohistiocytosis

## Abstract

Hemophagocytic lymphohistiocytosis (HLH) is a rare hyperinflammatory syndrome. Due to overlapping symptoms, primary HLH can be challenging to distinguish from brucellosis-associated HLH. This report describes the case of a 40-year-old male farmer who presented with progressive pancytopenia, body aches, fatigue, anorexia, weight loss, and fever. Laboratory examination showed severe leukopenia, anemia, thrombocytopenia, and abnormal liver function tests. Serology was positive for *Brucella, *but bone marrow culture was negative. Bone marrow biopsy showed histiocytic hemophagocytosis and preserved megakaryocytes. This confirmed HLH. Autoimmune and septic workup were unremarkable. Initially, the patient was suspected of having brucellosis-associated HLH, and anti-*Brucella* therapy was started, but cytopenia persisted. HLH-directed therapy was initiated. Treatment with dexamethasone and intravenous immunoglobulin resulted in hematological and clinical improvement. After thorough exclusion of infections and autoimmune disorders, a working diagnosis of primary HLH was established. The patient was discharged on oral corticosteroids with continuation of anti-*Brucella *therapy and scheduled chemotherapy. The patient showed continuous improvement after three cycles. This case shows the diagnostic complexity of HLH mimicking infection-associated syndromes.

## Introduction

Hemophagocytic lymphohistiocytosis (HLH) is a rare hyperinflammatory syndrome. This syndrome leads to cytokine storm due to overactivation of cytotoxic T‐cells, natural killer (NK) cells, and macrophages [1]. Activated macrophages carry out hemophagocytosis, which is the engulfment of blood cells by activated macrophages. Multiorgan failure and damage are caused by hypercytokinemia. There are two main forms of HLH, including genetic (primary) and acquired (secondary) [2].

Autosomal recessive immune deficiency is caused by biallelic mutations in genes regulating lymphocyte cytotoxic function (*PRF1*, *UNC13D*, *STX11*, or *STXBP2*) [3,4]. The *PRF 1* and *UNC13D* genes account for 40-60% of the mutations [5]. Although familial HLH classically presents in infancy, late presentations into adolescence or adulthood are recognized. Viral infections, immunodeficiency conditions, autoimmune diseases, and malignancies are the most common causes of secondary HLH. For example, Epstein-Barr virus (EBV) is among the most common HLH triggers [6]. Brucellosis is a zoonotic infection caused by *Brucella* species, transmitted to humans via contact with infected livestock or consumption of unpasteurized dairy products. It typically causes undulant fever, arthralgias, and hepatosplenomegaly, and frequently produces hematologic abnormalities such as anemia, leukopenia, and thrombocytopenia. In rare cases, brucellosis can trigger a secondary HLH. *Brucella*-associated HLH is exceedingly uncommon but often severe [7]. In endemic regions or at-risk individuals, clinicians must maintain a high index of suspicion for HLH in brucellosis.

Diagnosing HLH in adults remains challenging because its clinical and laboratory features often overlap with severe infections, autoimmune diseases, and malignancies. Delayed recognition may lead to multiorgan failure and poor outcomes. Furthermore, distinguishing primary from secondary HLH in adults is particularly difficult, especially in resource-limited settings where molecular testing may not be readily available. We report a unique case of an adult patient where our initial diagnosis was secondary HLH due to Brucellosis, but clinical course and extensive workup ultimately pointed to primary HLH.

## Case presentation

A 40-year-old male farmer was referred due to pancytopenia, generalized body aches, fatigue, anorexia, significant weight loss of about 10 kgs over weeks, poor oral intake, and dyspnea associated with physical activity. Patient described generalized body aches as a dull, aching pain involving multiple muscle groups diffusely. The pain was symmetrical, non-localized, and affected both proximal and distal areas of the body. Symptoms developed gradually and have been progressive over time. The pain was not related to physical exertion or trauma. There was also a complaint of intermittent subjective fever, described as on-and-off episodes over the course of the illness and resolved spontaneously or with the use of antipyretics. The systemic history of the patient was not significant. He was also not allergic to any medication or food. There was no family history of malignancy, autoimmune disease, hematological disorders, recurrent infections, or inherited conditions. The patient reported frequent exposure to farm animals.

On examination, he appeared ill and cachectic, with a body mass index of approximately 15 kg/m^2^ (weight 45 kg, height 170 cm). He was conscious, alert, and oriented. Vital signs were stable, and systemic examination was unremarkable. Splenomegaly was seen. Laboratory reports revealed that the patient was suffering from pancytopenia with leukopenia, and it was progressive (WBC lowest value = 0.54x109/L), along with anemia (hemoglobin (Hb) lowest value = 8.7g/dL). He had severe thrombocytopenia. His liver and metabolic parameters were also significantly deranged. His aspartate aminotransferase (AST) was raised to 198 U/L, and alanine transaminase (ALT) was up to 215 U/L with an increased level of bilirubin. His triglycerides were also raised (1.64-1.74 mmol/L). His renal functions were normal, but electrolyte disturbances like hyponatremia (Na = 126-129 mmol/L) and hyperkalemia (K = 5.83 mmol/L) were noticed. C-reactive protein (CRP) was raised (3.2-3.7 mg/L), and ferritin was markedly elevated (>3300 ng/mL), but it went down after treatment. Our patients’ laboratory reports further showed normocytic normochromic anemia with reticulocytosis but normal haptoglobin. Folate was markedly decreased (0.0 ng/mL), and iron was also low in blood (Table 1). The patient’s HScore was 167, supporting a high clinical probability of HLH and strengthening the diagnosis in the appropriate clinical context.

**Table 1:** Laboratory investigations AST: aspartate aminotransferase; ALT: alanine aminotransferase; CRP: C-reactive protein

Parameters	Patient Values	Reference Range
White blood cell count (lowest)	0.54 × 10⁹/L	4.0-11.0 × 10⁹/L
Hemoglobin (lowest)	8.7 g/dL	13.0-17.0 g/dL
AST	198 U/L	0-40 U/L
ALT	215 U/L	0-41 U/L
Triglycerides	1.64-1.74 mmol/L	<1.7 mmol/L
Serum sodium	126-129 mmol/L	135-145 mmol/L
Serum potassium	5.83 mmol/L	3.5-5.1 mmol/L
CRP	3.2-3.7 mg/L	<3.0 mg/L
Ferritin	>3300 ng/mL	30-400 ng/mL
Folate	0.0 ng/mL	3.0-17.0 ng/mL

**Figure 1:**
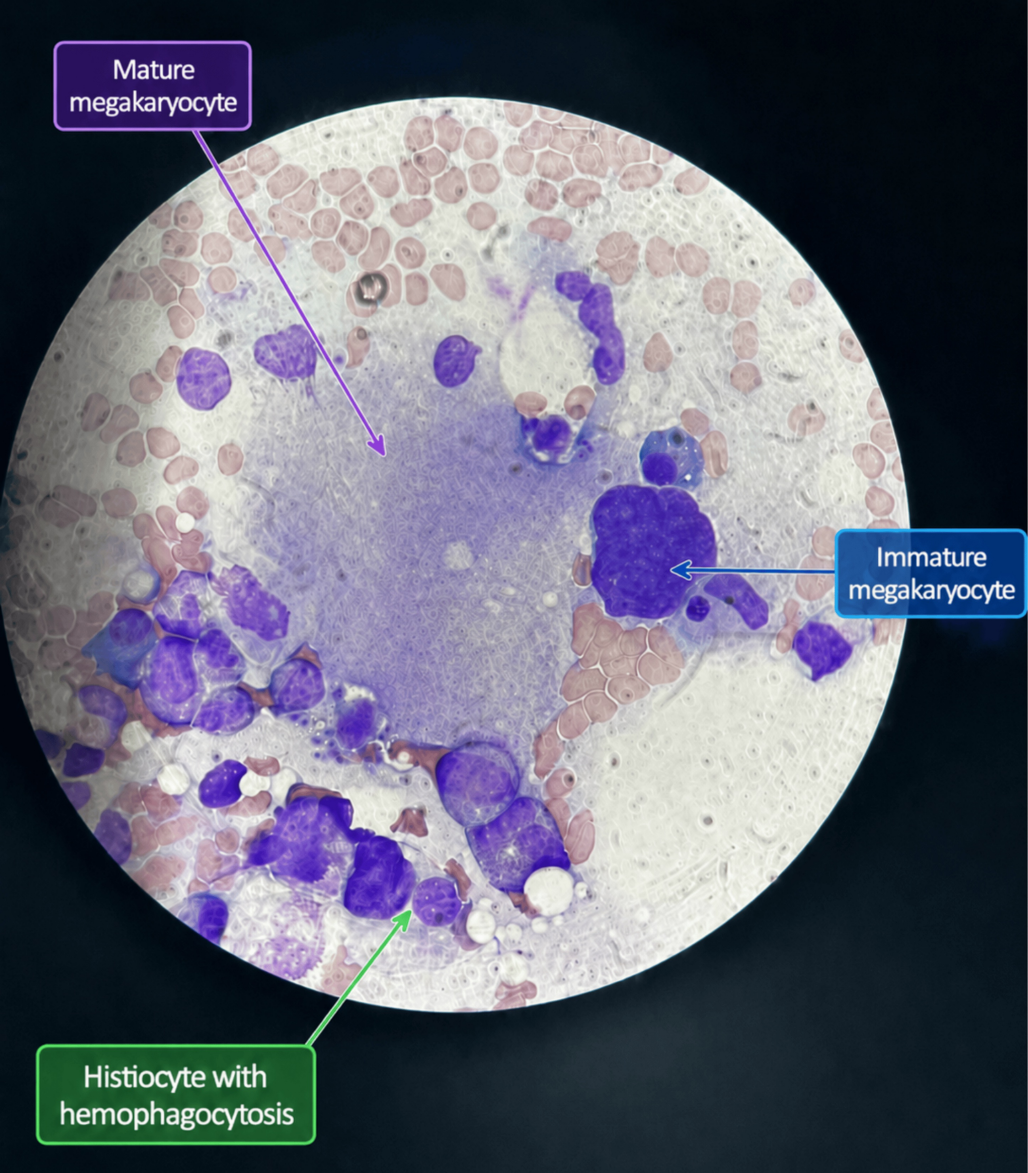
Bone marrow biopsy findings demonstrating hemophagocytosis. The bone marrow biopsy demonstrates a mature megakaryocyte (purple pointer) and an immature megakaryocyte (blue pointer). In addition, a histiocyte with hemophagocytosis (green pointer) is identified, showing engulfment of hematopoietic elements. These findings, together with the patient’s pancytopenia, fever, hyperferritinemia, and elevated liver enzymes, support the diagnosis of HLH. HLH: hemophagocytic lymphohistiocytosis

Serological testing for *Brucella *was positive (titer 1:320); however, bone marrow culture showed no growth. Based on the positive serology, secondary HLH was initially suspected. Bone marrow biopsy revealed both mature and immature megakaryocytes. Furthermore, a prominent number of histocytes were found with hemophagocytosis (Figure 1).

Autoimmune workup, including antinuclear antibody (ANA), anti-dsDNA, extractable nuclear antigen (ENA), and antiphospholipid antibodies, was negative except for a mildly elevated cancer antigen (CA)-125 level (51.3 U/mL). Septic screening was unremarkable, suggesting a non-bacterial hyperinflammatory process. Peripheral blood smear and bone marrow findings, together with pancytopenia, hyperferritinemia, elevated liver enzymes, and fever, supported the diagnosis of HLH. 

Given the mildly elevated CA-125 level and the possibility of malignancy-associated HLH, contrast-enhanced CT imaging of the chest, abdomen, and pelvis was performed. The scan demonstrated well-aerated lungs without pulmonary masses, consolidation, or interstitial disease. No mediastinal, hilar, abdominal, or pelvic lymphadenopathy was identified, and there was no evidence of hepatosplenic focal lesions, organ infiltration, ascites, or osseous lesions. Overall, CT findings were unremarkable with no radiologic evidence suggestive of underlying lymphoma or other malignancy.

During hospitalization, the patient was initially managed with intravenous fluids and proton pump inhibitors. Empirical anti-*Brucella* therapy (doxycycline 100 mg orally twice daily and streptomycin 1 g IM/IV daily) was administered for four days.

The patient remained clinically stable and afebrile, but cytopenias persisted, prompting consultation for hematology and further checkup for secondary HLH. Filgrastim was given to him to relieve his neutropenia, after which bone marrow aspiration and biopsy revealed hemophagocytosis. So, dexamethasone was started for HLH, and because it was responding partially, intravenous immunoglobulin (IVIG) (0.5 g/kg) was added. This led to marked improvement in our patient. Although no genetic testing was performed, after excluding other causes of HLH, the diagnosis of primary HLH was made. The patient was discharged in stable condition on oral dexamethasone along with continuation of anti-*Brucella* therapy. He was scheduled for outpatient chemotherapy and has since completed three cycles of etoposide with continued clinical improvement.

## Discussion

Clinical manifestation of HLH varies greatly. However, a common hallmark of this condition is fever associated with multiple organ involvement or their failure. In adults, there is still no clear consensus on standardized diagnostic criteria. The Histiocyte Society established diagnostic criteria for pediatric patients in 2004 [8]; however, these criteria are often applied to adults in clinical practice (Table 2). Later, an updated set of diagnostic guidelines was proposed in 2009 (Table 3) [9].

**Table 2:** Diagnostic criteria for HLH (2004) Hb: hemoglobin; TG: triglycerides; HLH: hemophagocytic lymphohistiocytosis Note: The table was independently created by the authors using information summarized from Henter et al., 2007 [8].

Criteria	Description
Definitive Diagnosis	Molecular or genetic diagnosis aligning with HLH
Other diagnostic approach	If five or more than five out of eight criteria listed below are present
Fever	Recurrent fever
Splenomegaly	Radiological or clinically large spleen
Cytopenias	Peripheral blood film shows presence of two or more than two blood lines
	Hb less than 9g/dl
	Absolute neutrophil count is less than 1x109/L
	Platelet less than 100x109/L
Hypertriglyceridemia/ hypofibrogenemia	Fasting TGs equal or more than 3mmol/L (greater than or equal to 265 mg/gl) and or fibrogen is less than or equal to 1.5 g/L
Hemophagocytosis	Bone marrow, lymph nodes or spleen provides evidence
Natural killer cell activity	Markedly decreased or absent
Level of ferritin	Greater than or equal to 500 microgram per liter
Soluble interleukin-2 receptor (sCD25)	Greater than or equal to 24,000 U/L

**Table 3:** Proposed HLH diagnostic criteria (2009) HLH: hemophagocytic lymphohistiocytosis Note: The table was independently created by the authors using information summarized from Filipovich, 2009 [9]

Category	Criteria
1. Molecular Diagnosis	HLH diagnosis confirmed on a molecular (genetic) basis
2. Clinical Criteria (≥3 required)	- Fever - Splenomegaly (enlarged spleen) - Cytopenia - Hepatitis
3. Laboratory/Immunologic Criteria (≥1 required)	- Elevated ferritin - Increased interleukin-2 receptor (sCD25) - Absent or decreased natural killer (NK) cell function
4. Supportive Findings	- Hypertriglyceridemia - Hyponatremia - Hypofibrinogenemia

Fardet et al. developed the HScore to estimate the probability of HLH in adult patients. The score incorporates a combination of clinical and laboratory parameters, including fever, organomegaly, ferritin level, triglycerides, ALT, immunosuppression, cytopenias, and hemophagocytosis on bone marrow examination. An HScore ≥250 corresponds to an approximate 99% probability of HLH, whereas a score ≤90 is associated with a probability of less than 1% [10]. Similarly, a study by Knaak et al. reported that among a cohort of 2,623 critically ill adults with hyperferritinemia, an HScore cutoff of 168 demonstrated excellent diagnostic performance, with 100% sensitivity and 94.1% specificity for HLH [11]. Furthermore, increasing HScore values were significantly associated with higher in-hospital mortality (OR 1.011, 95% CI 1.009-1.013; p<0.001). Five of the eight criteria, that is, fever, cytopenia, raised level of ferritin, elevated levels of triglycerides, and bone marrow hemophagocytosis, were fulfilled by our patient. Although positive *Brucella* serology and occupational exposure initially suggested brucellosis-associated secondary HLH, persistent pancytopenia and hyperferritinemia despite appropriate antibiotic therapy argued against infection alone as the underlying cause. Marked clinical and hematologic improvement following dexamethasone, IVIG, and etoposide, along with recurrence of cytopenias during steroid tapering, supported a diagnosis of probable primary HLH despite the absence of genetic confirmation.

Multiorgan failure is quite common in HLH. A key pathological feature is hemophagocytosis, in which activated macrophages engulf hematopoietic cells within the bone marrow [12]. Pulmonary involvement has been reported but remains relatively uncommon. A study involving 219 patients reported that almost 54% patients had lung involvement. Severe dysfunction of multiple organs was seen in the patients with lung involvement [13]. In contrast, our patient demonstrated well-aerated lungs with no radiological evidence of significant alveolar or interstitial disease. Although brucellosis is a common zoonotic infection, it is a rare trigger of secondary HLH. The diagnostic challenge arises from overlapping clinical features, as brucellosis can itself cause splenomegaly and cytopenias through involvement of the reticuloendothelial system [14]. In this case, the patient’s occupational exposure as a farmer and history of animal contact initially raised suspicion for brucellosis. Empirical anti-*Brucella* therapy was initiated; however, no clinical improvement was observed after four days, which prompted consideration of alternative diagnoses while continuing antibiotic therapy for a total of two weeks. Following suspicion of HLH, treatment with corticosteroids and IVIG was started, which resulted in initial clinical improvement. However, the patient developed recurrent pancytopenia upon tapering and discontinuation of steroids.

The initial step in treating HLH is to address the underlying cause, which could be a cancer, autoimmune disease, or viral infection. According to the HLH-94 protocol, etoposide induction therapy is recommended in cases of malignancy-associated HLH or when clinical worsening is evident. Cyclosporin, IV immunoglobulin, and corticosteroids are further treatments. Only refractory cases are eligible for allogenic stem cell transplantation [15,16]. After a full course of antibiotics for brucellosis and exclusion of other alternative differential diagnoses, we started etoposide (three cycles until now), and he showed improvement.

## Conclusions

This case highlights the diagnostic and therapeutic complexity of HLH, particularly when it presents with features overlapping infectious conditions such as brucellosis. In this case, patient received treatment adequately for brucellosis and apparently met the requirements for secondary HLH but the case was reexamined since pancytopenia, hyperferritinemia, and transaminitis persisted despite antibiotic therapy. Although no genetic testing was done but remarkable response to treatment with corticosteroids, IVIG, and etoposide strongly suggested probable primary HLH. This emphasizes the actual fact that HLH might need to be treated based on responses to therapy and elimination of additional factors rather than only confirmation on molecular basis in places with inadequate resources.
